# Weight management personas of breast cancer patients undergoing chemotherapy in China: a multi-method study

**DOI:** 10.1186/s12911-024-02515-1

**Published:** 2024-04-25

**Authors:** Xinyu Li, Nan Zhang, Juan Yang, Zhaohui Geng, Jie Zhou, Jinyu Zhang

**Affiliations:** 1 Shanghai University of Traditional Chinese Medicine, Shanghai, China; 2 Ruijin Hospital, Shanghai Jiaotong University School of Medicine, Shanghai, China; 3 Yueyang Hospital of Integrated Chinese and Western Medicine, Shanghai University of Traditional Chinese Medicine, Shanghai, China

**Keywords:** Breast cancer, Weight management, Persona, Chemotherapy, mHealth

## Abstract

**Background:**

Mobile health (mHealth) may be an ideal solution for breast cancer (BC) patients in China to access weight management interventions. User retention and engagement are the main challenges faced by mHealth applications. A user persona, which is a user-centered design process, can lead to the development of mHealth that is more acceptable to the needs of target users. This study aimed to investigate the variety of experiences in weight management and the behavioral preferences of BC patients receiving chemotherapy to develop users’ personal information and persona development for the design and implementation of mHealth interventions.

**Methods:**

Sixteen individual semi-structured in-depth interviews were conducted with BC patients receiving chemotherapy. We employed the thematic analysis method to analyze the interview transcripts in NVivo 11 software. The themes obtained from the analysis were used as the subdomains of personas. A proforma was designed to extract each participant’s experience in each subdomain. Patients who exhibited similar experience in subdomains were grouped into a persona using affinity diagrams. The personas were named according to their prominent features. A questionnaire survey was conducted to validate the personas and to test whether the personas that were generated from the qualitative interview data were applicable to the Chinese population with BC.

**Results:**

Four themes were identified as subdomains of weight management personas: the perception of weight management while undergoing chemotherapy, symptoms and emotional disturbance, changes in diet and exercise, and health literacy and information seeking. Five personas were ultimately obtained: (1) positive weight controllers, (2) patients who were inactive due to fatigue, (3) young patients who avoided communication, (4) overweight patients with treatment priority, and (5) patients who engaged in irregular exercise. Finally, the quantitative study showed that 51.58% of patients chose one of these five personas to represent themselves in weight management. None of the patient reported selecting options that were not explicitly outlined in the questionnaire and provided personalized descriptions of their weight management characteristics.

**Conclusions:**

The selected personas were developed from in-depth interviews on biopsychosocial areas. They highlight different weight management patterns in Chinese BC patients and provide implications for both the design of mHealth systems and traditional interventions.

**Supplementary Information:**

The online version contains supplementary material available at 10.1186/s12911-024-02515-1.

## Introduction

Mobile health (mHealth), which has the ability to deliver health care through applications on mobile devices, is an approach that can bring about meaningful healthcare change [1]. Convenient, accessible, and low-cost mHealth apps for health management are becoming popular and indispensable in China because they can meet the various needs of patients and alleviate the shortage of medical resources in some rural areas [2–4]. However, the main challenge for mHealth apps is user retention and attraction [5–8], and the key to improving these aspects is to design the apps with a user-centered design (UCD) approach.

User personas are hypothetical archetypes that represent groups of targeted users to enable technology developers to achieve a deep and intuitive understanding of numerous and diverse target users [9–11]. As a classical UCD tool, user personas are widely used in product marketing fields. Personas that are applicable to the health management field go well beyond demographics as they attempt to capture patients’ mental models, which comprise their expectations, prior experience and anticipated behavior [12]. Personas, especially those that include biopsychosocial data, are also key references for patient classification and personalized intervention. In the context of mHealth, biopsychosocial personas represent a distinctive category of personas that comprehensively incorporate physical, psychological, and social attributes as well as user goals, and all features of personas are related to maintaining health or recovering from disease [12–14]. The design and delivery of personalized information or interventions based on different personas can further improve the experience of mHealth users by providing them with the tailored help that they need most [13], which can increase user retention and engagement. Personas can be used as part of an overall UCD approach or can augment existing approaches by bringing UCD thinking into the design and development of mHealth.

Despite their potential benefits, the use of personas has not received considerable attention in the design and development of mHealth services and interventions. In the literature, only a few reference studies that talk about the development of patients’ personas in the fields of online health communities [13], home health care technologies [15], patient decision-making [16] and adherence to cardiovascular diseases medication [12]. There is no consensus on a single way of constructing personas and matching them with the actual users. However, some options include the use of qualitative thematic analysis [17] or cluster analysis [14] of quantitative data or mixed methods [13]. The personal data obtained through qualitative interviews are in-depth and comprehensive [12] and thus are especially suitable for primary exploratory research.

Breast cancer (BC) remains the most common cancer in women [18]. There were 306,000 new cases of BC in 2016, accounting for 16.72% of all new cancer cases in China [19]. Obesity and treatment-induced weight gain are well documented among this population. The prevalence of overweight and obesity among BC patients is approximately 50% [20, 21]. A meta-analysis of 25 articles and 2620 participants showed an average weight increase of 5.95 lb (95% CI: 2.0 to 7.5) during chemotherapy [22]. Previous systematic reviews and clinical trials have shown that being overweight or obese before or shortly after diagnosis [23, 24] as well as the occurrence of weight gain after diagnosis [25] are associated with increased risks of BC-specific mortality, all-cause mortality, and recurrence. Therefore, weight management among BC survivors aimed at maintaining a healthy body weight is recommended by published guidelines [26–28] in various countries.

Weight management interventions rely on a three-part approach, namely, diet, exercise, and behavioral therapy [29]. The intervention effects are influenced by social support [30], health relief [31], and chemotherapy-related symptoms, particularly fatigue, which is a key reason for exercise abandonment. The behavioral preferences and different experiences of BC patients undergoing chemotherapy require consideration of user diversity and its categorization as critical factors. These factors should be carefully considered when developing mHealth weight management applications. Although several weight management mHealth apps or programs have been developed, few studies have described the personas of the targeted users.

Considering the complexity and heterogeneity of individual weight management [32, 33], these personas could be a powerful tool for understanding users’ preferences and initial needs. This study aimed at developing user personas that illustrate the variety of experiences in weight management and the behavioral preferences of BC patients receiving chemotherapy. It also aims to propose paths for the use of personas in the design and implementation of mHealth interventions.

## Methods

### Sampling and recruitment

The study took place in China and involved a qualitative study that applied in-depth interviews and a short questionnaire. Initially, a qualitative study was conducted between September and December 2021. Subsequently, the persona development was completed, and a questionnaire was administered between June and October 2022 to validate and corroborate the findings from the qualitative study. The study maintained consistent inclusion and exclusion criteria across its two phases, recruiting participants from the same BC chemotherapy clinic of a 3 A hospital in Shanghai. This approach aimed at facilitating a thorough exploration of weight management personas among BC patients undergoing chemotherapy within a homogenous target population. All participants were recruited if they were (1) BC patients receiving chemotherapy or within one month of completing chemotherapy, (2) female, (3) aged ≥ 18 years, and (4) proficient in the use of smartphone software. Individuals with advanced BC, those who had not finished the initial course of chemotherapy, or those who had other severe underlying diseases were excluded from enrollment.

### Data collection

The BC patients in the sample were interviewed using a semi-structured interview approach that involved probing questions such as, “What do you think about weight management during the period of chemotherapy?” and “What do you do to maintain a healthy weight?” This method allowed for a nuanced exploration of the participants’ experiences and concerns about weight management. The main content of the interviews included individual characteristics, mobile device usage and preferences and their current status of weight management. To ensure focus and relevance, if interviewees deviated from the central theme, the interviewers adeptly guided the conversation back to the predetermined topics of inquiry. The interviews were conducted in person by well-trained interviewers with experience in qualitative interviewing at places chosen by each participant. Informed consent regarding participation and recording was obtained before the interviews using a participant consent form and information sheet. Participants could refuse to answer any of the questions and/or discontinue participation in the research at any time.

After obtaining the consent of the interviewee, the interview process was audio recorded. Moreover, the interviewer carefully observed and recorded the nonverbal behaviors of the interviewee during the interview. Each participant was interviewed for approximately 30 to 50 min. The interviews continued until no new information emerged and information saturation was reached.

To assess the validity of the personas, a concise questionnaire focused on weight management personas was subsequently administered. This instrument aimed to ascertain the most fitting persona for each participant based on the participant’s current condition. The questionnaire was designed to systematically incorporate the data obtained from the interviews into a structured survey format. It included sociodemographic inquiries and integrated findings derived from the preceding qualitative study. This integration involved the incorporation of five personas as response options, each accompanied by detailed information on thematic elements identified in the qualitative investigation. The condition of participants may not coincide entirely with any single persona; hence, we permitted participants to select up to two options that best corresponded with their health status. (Appendix 1).

### Data analysis

#### Themes analysis

**Fig. 1:**
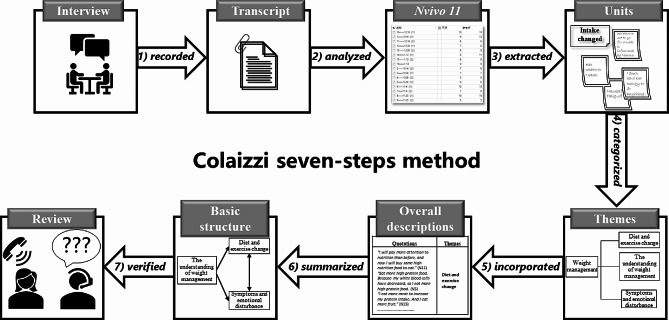
Procedure of the Colaizzi method

Within 24 h of the interview, the audio files were transcribed verbatim. NVivo 11 software was used to conduct a thematic analysis based on Colaizzi’s method [34]. The steps were as follows: (1) interview recordings were listened to carefully, and the participants’ words were recorded verbatim to reflect the complete content of the interview and to gain a holistic understanding of the participant’s experience; (2) the verbatim transcripts were entered into NVivo11 and analyzed qualitatively as meaningful sentences; (3) meaningful units were extracted from the meaningful sentences; (4) common characteristics of the identified meaningful units were clustered and categorized into different themes; (5) the themes of the research were incorporated to organize more detailed overall descriptions; (6) detailed descriptions were produced to further capture and present the basic structure of the patients’ experiences; and (7) the participants were invited to review the analyzed data to verify whether the analysis reflected their authentic experience (Fig. 1).

### Persona development

The subdomains of personas were designed based on themes that emerged from the qualitative research analysis. A pro forma was meticulously designed to summarize all participants’ past experiences with weight management across the subdomains as well as their demographic and mobile phone usage information. First, patients with similar experiences in certain subdomains were grouped into personas using affinity diagrams. Their demographic and mobile phone usage characteristics were subsequently added to enhance the personas. Finally, the personas were named according to their prominent features. To maintain focused attention on the behaviors and characteristics of the user population in China, capital letters and names were used to represent the personas rather than specific avatars.

### Persona validation

SPSS software version 24.0 was used to analyze the demographic and clinical data as descriptive statistics. The amount and proportion of the options containing all possible combinations were also calculated.

### The Population context of the qualitative interview participants

A total of 16 interviews were conducted. The participant characteristics are outlined in Table 1.

**Table 1:** Characteristics of the participants

No	Age (years)	BMI (kg/m^2^)	Educational level	Employment	Current treatment
N1	37	20.20	College	Sick leave	Targeted therapy
N2	41	27.61	High school	Sick leave	Postoperative chemotherapy; targeted therapy
N3	39	25.40	College	Sick leave	Targeted therapy
N4	41	26.45	College	Sick leave	Postoperative chemotherapy
N5	44	18.82	Postgraduate	Employed	Postoperative chemotherapy
N6	40	23.62	College	Sick leave	Metastatic chemotherapy; targeted therapy
N7	50	22.49	College	Sick leave	Post-recurrence chemotherapy; endocrine treatment
N8	63	22.83	College	Retired	Postoperative chemotherapy
N9	30	18.37	Postgraduate	Student	Postoperative chemotherapy; targeted therapy
N10	45	22.03	High school	Sick leave	Neoadjuvant chemotherapy
N11	50	19.31	High school	Retired	Metastatic Chemotherapy; targeted therapy
N12	44	22.66	Postgraduate	Sick leave	Postoperative chemotherapy; Chinese medicine
N13	40	23.42	High school	Sick leave	Metastatic Chemotherapy; targeted therapy
N14	52	28.58	High school	Retired	Postoperative chemotherapy
N15	47	24.80	High school	Sick leave	Postoperative chemotherapy; targeted therapy
N16	21	22.27	College	Student	Postoperative chemotherapy; targeted therapy

### Themes and detailed statements of participants

Four themes concerning the weight management of BC patients during chemotherapy were revealed through the interviews. They included the perception of weight management while undergoing chemotherapy, symptoms and emotional disturbances, changes in diet and exercise and health literacy and information seeking. Participants’ quotations are presented in Table 2 to support these themes.

**Table 2:** Themes and Participant quotations

Quotations	Themes
*“I ain’t got no fancy knowledge about managing weight, but I do know one thing for sure: packing on pounds ain’t good news, especially when you’re dealing with chemo. Heard from another patient that weight gain comes with the territory, so gotta watch what you eat. But why? That’s still a mystery to me.”* (N15) *“I never gave a darn about my weight and never even crossed my mind to bother.”* (N3) *“I weigh myself once a week, but I am more concerned about the treatment.”* (N2)	The perception of weight management while undergoing chemotherapy
*“I’m just not feeling hungry at all, can’t even force myself to eat, feels like those pregnancy days when food was a no-go. Not feeling the urge to chow down. Plus, I’m kinda queasy too.”* (N10) *“My muscles were just plain weak all over.”* (N11) *“My body feels like it’s on empty, real weak. Sometimes I’m just counting down the minutes till I can crash cause every time I’m up, I’m tossing my cookies.”* (N3) *“It is a negative experience. I’m afraid of death. The more that I know, the heavier the burden I have.”* (N13) *“I’m feeling old and out of touch. Ain’t in the mood to chat with anyone. Can’t seem to find any zest for life. Nothing makes me unhappy nor happy.”* (N8)	Symptoms and emotional disturbance
*“I will pay more attention to nutrition than before, and now I will buy some high-nutrition food to eat.”* (N11) *“Gotta chow down on more protein-rich grub. My white blood cells are taking a nosedive, so loading up on the good stuff.”* (N5) *“I’m beefing up on meat for that protein boost. Also, I’m loading up on more fruit.”* (N15) *“I’ve been keeping it light with home-cooked meals and cutting down on takeout.”* (N9) *“I hit the jump rope 1 or 2 times a week, knocking out about 3000 jumps each session.”* (N12) *“I’m all about that cardio life. I’m following this Keep app at home for jump fitness and stretching routines, hitting it up 3–4 times a week.”* (N7) *“I do not exercise during chemotherapy.”* (N10)	Diet and exercise change
*“I read and study the literature recommended by hospitals or doctors.”* (N6) *“I’m part of this WeChat crew for breast cancer fighters, where we swap stories, tips, and all that jazz.”* (N1) *“The rehab doc gave me the green light to start working out, so I dove in and slowly cranked up the intensity bit by bit.”* (N5) *“We do whatever the doctor says.”* (N9) *“I’ve been googling stuff online, trying to figure out what foods are gonna do me good.”* (N15) *“I’m hooked on this hospital and doctor’s WeChat page. They shoot out these little articles about different sicknesses.”* (N14)	Health literacy and information seeking

### User personas for weight management while undergoing chemotherapy

Five personas were produced by systematically collating and analyzing the verbatim transcripts (Table 3). The participants were identified by capital letters for confidentiality purposes, and quotes from 16 qualitative interviews were used. The implications and recommendations are presented in Table 4.

**Table 3:** Weight Management Personas Overview

Subdomains	Persona A Positive weight controllers	Persona B Patients who were inactive due to fatigue	Persona C Young patients who avoided communication	Persona D Overweight patients with treatment priority	Persona E Patients who engaged in irregular exercise
**Representative figures**	N5-N7	N10-N12	N9, N16	N2-N4, N8, N14	N1, N13, N15
**Age**	40–50	44–50	21–30	39–63	37–47
**Education level**	College or above	High school or above	College or above	High school or college	High school or college
**BMI**	Normal (18.82 ~ 23.62 kg/m^2^)	Normal (19.31 ~ 22.66 kg/m^2^)	Normal (18.37 ~ 22.27 kg/m^2^)	Overweight or gained weight after diagnosis (22.83 ~ 28.58 kg/m^2^)	Normal (20.20 ~ 24.80 kg/m^2^)
**Chemotherapy cycle**	7–8	2–3	2–6	7–8	2–8
**Daily mobile phone time**	≤ 4 h	4–6 h	>6 h	4–6 h	>6 h
**Perception of weight management while undergoing chemotherapy**	They are knowledgeable about weight management and are proactive in managing their weight.	They do not know about weight management.	They do not understand weight management and are afraid of knowing too much	They do not understand or know a little about weight management as a treatment priority	They know a little about weight management and control their BMI in the normal range
**Symptoms and emotional disturbance**	Mild symptoms: nausea, vomiting and fatigue No emotional issues	Severe fatigue No emotional issues	Nausea and vomiting Avoiding any kind of contact	Severe symptoms, particularly nausea High mental burden	Fatigue No emotional issues
**Diet and exercise change**	Regular exercise, both before and after diagnosis	Inactive due to fatigue: no exercise habit or a dislike of sports	No exercise to occasional walking	No exercise	Irregular exercise, with no exercise habits before diagnosis
	High-protein diet, increased vegetable and fruit intake and limited total calorie intake	A nutrition-focused diet with food for elevated white blood cells	Intake of more vegetables and better nutrition after diagnosis	Unrestricted diet	A high-protein diet and more vegetables and fruits
**Health literacy and information seeking**	Actively seeking knowledge, such as questioning rehabilitation doctors or reading professional literature	Network resources and communication with other patients	None or just communication with other patients	Network resources and communication with other patients	Network resources and communication with other patients

**Table 4:** Persona-Tailored Weight Management Recommendations

Personas	Recommendations
**Persona A-The positive weight controller**	-provide professional counseling services -develop personalized weight management plans with patients according to their habits and preferences -evaluate the effect of weight management
**Persona B-The patient who were inactive due to fatigue**	-explain the positive effect of exercise on fatigue symptoms -encourage and instruct patients to choose appropriate exercise according to their health conditions -for patients with serious fatigue and poor physical strength, the intensity of exercise should be reduced according to need, step by step, and the amount and intensity of exercise should then be gradually increased
**Persona C-Th young patient who avoided communication**	-priority should be given to solving psychological problems, changing negative coping styles, and transforming emotional coping styles from problematic coping style -young breast cancer fellowship platforms should be built to share anticancer experiences and provide support to each other
**Persona D- The overweight patient with treatment priority**	- teach patients about the importance of weight management during the chemotherapy period -chemotherapy-related symptom management should be carried out to control the impact of adverse chemotherapy reactions in treatment and daily life -encourage patients to gradually engage in physical activity, follow a healthy diet and maintain a healthy BMI during chemotherapy
**Persona E- The patient who engaged in irregular exercise**	-choose favorite exercise and create an exercise plan with patients -urge patients to exercise regularly every day

### Persona validation

To validate and extend the insights obtained from the qualitative phase, a questionnaire was designed and administered with the same inclusion and exclusion criteria for participant selection as the interview. A total of 95 BC patients who were receiving chemotherapy participated in the survey. The characteristics of all participants and each persona are displayed in Table 5. The average age was 55.20 ± 12.10 years, and the average BMI was 22.98 ± 2.31 kg/m^2^. The results are displayed in Table 6. Of the participants, 49 of 95 (51.58%) chose one of five personas to represent themselves in weight management. Some participants (48.42%) thought that two different personas were relevant to them. Most of the participants selected Persona A (21.05%), while the combination of Persona B and Persona D was chosen by others (13.68%). None of the participants simultaneously chose Persona A and Persona E.

**Table 5:** Participant characteristics of questionnaire survey by persona

Variables	All (*n* = 95)	Persona A (*n* = 27)	Persona B (*n* = 36)	Persona C (*n* = 25)	Persona D (*n* = 35)	Persona E (*n* = 18)
Age, years: Mean ± SD	55.20 ± 12.10	53.82 ± 13.56	59.16 ± 11.07	50.42 ± 13.92	60.1 ± 9.42	56.05 ± 9.10
Time of diagnosis, years: Mean ± SD	5.62 ± 12.07	3.21 ± 2.02	3.64 ± 2.72	7.41 ± 21.22	8.62 ± 19.42	7.03 ± 9.76
BMI, kg/m^2^: Mean ± SD	22.98 ± 2.31	22.83 ± 2.39	23.26 ± 2.44	22.89 ± 2.27	23.37 ± 1.96	22.92 ± 2.72
Chemotherapy cycle: Mean ± SD	4.00 ± 2.24	3.20 ± 1.50	4.03 ± 2.47	3.70 ± 1.38	4.39 ± 3.03	4.09 ± 2.63
Ethnicity						
Han	94 (98.95%)	26 (96.30%)	36 (100%)	25 (100%)	35 (100%)	18 (100%)
Others	1 (1.05%)	1 (3.70%)	0 (0%)	0 (0%)	0 (0%)	0 (0%)
Educational level						
Primary school or below	9 (9.47%)	2 (7.41%)	6 (16.67%)	3 (12.00%)	3 (8.57%)	1 (5.56%)
Middle school	15 (15.79%)	3 (11.11%)	4 (11.11%)	4 (16.00%)	10 (28.57%)	2 (11.11%)
High school	34 (35.79%)	8 (29.63%)	16 (44.44%)	7 (28.00%)	11 (31.43%)	7 (38.89%)
College or above	37 (38.95%)	14 (51.85%)	10 (27.78%)	11 (44.00%)	11 (31.43%)	8 (44.44%)
Marital Status						
Married/ In a relationship	88 (92.63%)	25 (92.59%)	34 (94.44%)	22 (88.00%)	35 (100%)	17 (94.44%)
Single / Divorced / Widowed	7 (7.37%)	2 (7.41%)	2 (5.56%)	3 (12.00%)	0 (0%)	1 (5.56%)
Residence area						
City	82 (86.32%)	20 (74.07%)	33 (91.67%)	22 (88.00%)	32 (91.43%)	17 (94.44%)
Town	9 (9.47%)	6 (22.22%)	2 (5.56%)	1 (4.00%)	1 (2.86%)	1 (5.56%)
Village	4 (4.21%)	1 (3.71%)	1 (2.77%)	2 (8.00%)	2 (5.71%)	0 (0%)
Employment status						
Employed	21 (22.11%)	7 (25.93%)	5 (13.89%)	12 (48.00%)	7 (20.00%)	5 (27.78%)
Sick leave/ Unemployed	23 (24.21%)	8 (29.63%)	8 (22.22%)	7 (28.00%)	8 (22.86%)	4 (22.22%)
Retired	51 (53.68%)	12 (44.44%)	23 (63.89%)	6 (24.00%)	20 (57.14%)	9 (50.00%)
Family income (RMB)						
< 3000	3 (3.16%)	0 (0%)	3 (8.33%)	2 (8.00%)	1 (2.86%)	0 (0%)
3000–5000	35 (36.84%)	8 (29.63%)	15 (41.67%)	7 (28.00%)	14 (40.00%)	5 (27.78%)
5000–10,000	45 (47.37%)	13 (48.15%)	16 (44.44%)	13 (52.00%)	18 (51.43%)	10 (55.56%)
>10,000	12 (12.63%)	6 (22.22%)	2 (5.56%)	3 (12.00%)	2 (5.71%)	3 (16.66%)
Health insurance						
Without health insurance	4 (4.21%)	0 (0%)	3 (8.330%)	0 (0%)	7 (20.00%)	0 (0%)
Rural program	1 (1.05%)	0 (0%)	0 (0%)	0 (0%)	1 (2.86%)	0 (0%)
Citizen program	62 (65.27%)	16 (59.26%)	27 (75.00%)	4 (16.00%)	26 (74.28%)	11 (61.11%)
Employee health insurance	27 (28.42%)	11 (40.74%)	6 (16.67%)	21 (84.00%)	1 (2.86%)	6 (33.33%)
Others	1 (1.05%)	0 (0%)	0 (0%)	0 (0%)	0 (0%)	1 (5.56%)
Granulocyte colony-stimulating factors (G-CSFs) used						
No	79 (83.16%)	25 (92.59%)	31 (86.11%)	21 (84.00%)	25 (71.43%)	13 (72.22%)
Yes	16 (16.84%)	2 (7.41%)	5 (13.89%)	4 (16.00%)	10 (28.57%)	5 (27.78%)
Chemotherapy type						
Neoadjuvant chemotherapy	30 (31.58%)	9 (33.33%)	12 (33.33%)	9 (36.00%)	5 (31.43%)	5 (27.78%)
Postoperative chemotherapy	65 (68.42%)	18 (66.67%)	24 (66.67%)	16 (64.00%)	24 (68.57%)	13 (72.22%)
Daily average screen time						
<4 h	9 (9.48%)	15 (55.56%)	4 (11.12%)	3 (12.00%)	4 (11.43%)	0 (0%)
4–6 h	33 (34.74%)	10 (37.04%)	16 (44.44%)	7 (28.00%)	17 (48.57%)	3 (16.67%)
>6 h	53 (55.78%)	2 (7.740%)	16 (44.44%)	15 (60.00%)	14 (40.00%)	15 (83.33%)

**Table 6:** The proportions of persona combinations (*n* = 95)

Persona A	Persona B	Persona C	Persona D	Persona E	Number
√					20 (21.05%)
	√				7 (7.37%)
		√			6 (6.32%)
			√		9 (9.47%)
				√	7 (7.37%)
√	√				4 (4.21%)
√		√			2 (2.21%)
√			√		1 (1.05%)
√				√	0
	√	√			8 (8.42%)
	√		√		13 (13.68%)
	√			√	4 (4.21%)
		√	√		7 (7.37%)
		√		√	2 (2.11%)
			√	√	5 (5.26%)
27 (28.42%)	36 (37.89%)	25 (26.32%)	35 (36.84%)	18 (18.95%)	

## Discussion

### Principal results

This is the first study to explore the weight management personas of BC patients receiving chemotherapy. Five weight management personas with distinct attributes were developed to reflect Chinese BC patients receiving chemotherapy. Each of the personas and their features have implications for structuring a weight management mHealth system with interventions (see Table 4).

In our study, most BC patients, except for those belonging to Persona A, lacked an accurate perception of weight management during chemotherapy. The importance of weight management for patients receiving chemotherapy should be emphasized, especially for those grouped under Persona D, overweight patients with treatment priority. Persona D represents a significant portion of the treatment-priority Chinese chemotherapy patients who do not mind being overweight or experiencing weight gain during chemotherapy. Weight management intervention for patients belonging to Persona C, young patients who avoid communication, should provide more emotional support to help these patients find a compromise with chemotherapy and learn to live with cancer. Previous studies have shown that younger BC patients need more support from family and society than older patients [35, 36] because they often have a more difficult time with family and social pressure during their treatment and rehabilitation and face issues such as childbirth, therapeutic menopause, and returning to work [37, 38]. While an increasing number of studies have confirmed that regular moderate exercise can alleviate cancer-related fatigue [25], patients who were included in Persona B avoided exercise due to severe fatigue. Patients in this group were often in the early stage of chemotherapy and lacked knowledge of weight management, especially the positive effect of moderate exercise on cancer-related fatigue. For patients who do not have regular exercise habits or did not exercise before their BC diagnosis (Persona E), the focus of weight management should be on selecting sustainable exercise routines, developing exercise plans, and encouraging daily exercise.

An increasing number of studies [39] have begun to report the efficacy of multicomponent-tailored interventions in improving the outcome of BC patients in the field of personalized care. To achieve a better intervention effect, the mHealth weight management system should incorporate different intervention programs that are tailored to each weight management persona. In this study, participants were sorted into different intervention programs based on the features of their selected persona. The weight management personas of BC patients receiving chemotherapy may develop further during the intervention process due to increasing understanding and aptitude regarding weight management. Therefore, the personas used in the mHealth system need to be re-evaluated in a timely manner and adapted and matched to the intervention modules of the new personas.

As mHealth becomes increasingly popular, health researchers need to consider UCD approaches, such as the creation of personas, to provide context-appropriate and personalized health interventions [14, 40]. Weight management personas of BC patients can be used to assist designers and health researchers in considering a variety of potentially relevant factors that may influence weight management when designing mHealth systems or interventions [41]. Studying user personas can provide insights into what user types should be considered for weight management mHealth interventions and the best ways to do so. The use of different personas also allows designers to accommodate all potential users rather than ignoring any particular group. Previous studies [17] have demonstrated that personas can be used to evaluate existing products by providing scenarios for identifying and challenging political and social assumptions about users. Personas can not only be used to guide the design of patient-centered mHealth systems and services but also to provide reference tools for the planning and implementation of traditional interventions by contributing to a full understanding of the relevant population. Haldane [12] argued that studying personas enables a middle path in highlighting user diversity and developing individualized health management systems in a manageable and actionable way. While it may not be realistic to design and apply mHealth interventions to match the preferences or weight management patterns of each individual, it is feasible and cost-effective to do so for each user group.

To date, there is no consensus on how to form user personas. Most related studies [11, 17, 42] have used qualitative interviews to develop user personas. Richard J. Holden [14] introduced a generic ten-step persona development process in a study of older adults with heart failure. Several studies have applied quantitative cluster analysis methods for persona development [43]. The advantages of this approach include efficiency in data collection and analysis and the ability to cluster individuals onto multiple variables simultaneously [14]. Quantitative approaches are limited by the lack of a deep understanding of each variable and how these variables relate to the specific information needs addressed by mHealth. In quantitative clustering analysis, it may be difficult for healthcare researchers to translate clusters into concrete meaningful designs without deeper and more contextualized knowledge of the relevant subdomain variables [14]. By comparison, a persona formed by qualitative thematic analysis is more subjective and imprecise but is also richer and provides more direct implications for mHealth design. While mixed-method persona development has been described in a few studies [12, 13], the way that qualitative interview and quantitative survey data should be combined has not yet been determined. The aim of our study was to explore and develop weight management personas to guide the design of mHealth services and traditional interventions so that more vivid personas can be generated through the use of in-depth interview data. The current consensus on user personas in health management is that these personas need to cover biopsychosocial aspects, but it is unclear what subdomains should be included in each specific persona and how they should be identified for different health management goals. Our study demonstrated that thematic analysis of qualitative interviews is a viable approach for identifying the subdomains of weight management roles. Although disparities existed in the sociodemographic characteristics of participants in the questionnaire survey and the personas identified through qualitative research, discernible trends were identified. For instance, Persona C, which was predominantly composed of young individuals in the qualitative study, exhibited a corresponding pattern in the questionnaire wherein the age distribution of Persona C was notably youthful and nearly half of the participants of Persona C were employed. This finding demonstrates the credibility and logical coherence of our validation survey and affirms its capacity to furnish supplementary reference information. A user persona could lack generalizability if it were derived from a small sample. Given the nature of the qualitative approach, some researchers argue that personas can be complemented by using quantitative analysis methods to verify the validation [14]. The validation of personas following persona development has been raised as a critique of persona research [44]. In our study, external validation was used to verify the usability of the personas. More than half of the participants found the right persona to represent themselves, and none failed to find a persona that matched them. This indicates that the weight management personas developed in our study are suitable for most people in China and cover all typical user archetypes without the need to add new weight management personas.

### Limitations

There are a few limitations to our study. First, the applicability of personas is limited by the fact that persona development was based on BC patients in China. The generalizability of these findings needs to be extended to different countries. Second, our study recruited only smartphone users. The number of BC patients who did not have access to the internet or were unable to use smartphones was marginal. Third, we did not track changes in personas throughout the entire chemotherapy period. The personas of participants could vary over time. Thus, future studies should focus on the longitudinal development of personas.

## Conclusions

Our study explores the creation and validation of biopsychosocial personas and how these personas highlight different weight management patterns. It also provides implications for the design of mHealth systems and traditional interventions. In this study, five different weight management personas were constructed and tested among BC chemotherapy patients based on differences in their perception of weight management while receiving chemotherapy, symptoms and emotional disturbances, diet and exercise changes, health literacy, and information seeking. Developing multidimensional personas through qualitative interviews to better understand the target population is a viable approach to guide the design of mHealth services and interventions.

### Electronic supplementary material

Below is the link to the electronic supplementary material.

Supplementary Material 1

## Data Availability

The datasets used or analyzed during the current study are available from the corresponding author on reasonable request.

## Abbreviations

BC: breast cancer
mHealth: mobile health
UCD: user-centered design
