# Correction: Weight management personas of breast cancer patients undergoing chemotherapy in China: a multi-method study

**DOI:** 10.1186/s12911-024-02532-0

**Published:** 2024-05-21

**Authors:** Xinyu Li, Nan Zhang, Juan Yang, Zhaohui Geng, Jie Zhou, Jinyu Zhang

**Affiliations:** 1 Shanghai University of Traditional Chinese Medicine, Shanghai, China; 2 Ruijin Hospital, Shanghai Jiaotong University School of Medicine, Shanghai, China; 3 Yueyang Hospital of Integrated Chinese and Western Medicine, Shanghai University of Traditional Chinese Medicine, Shanghai, China

**Correction to: Li et al. BMC Medical Informatics and Decision Making (2024) 24:108**

10.1186/s12911-024-02515-1

Following the publication of the original article, the authors reported that the Funding statement was incomplete. The published version only mentioned the fund from the 2020 Shanghai Health Commission Clinical Research Special (no. 202040487).

The complete funding note is as follows:

This study was supported by the 2020 Shanghai Health Commission Clinical Research Special (No. 202040487), Shanghai Science and Technology Innovation Action Plan Sailing Project (No. 21YF1447700), Humanities and Social Science Research Project of Ministry of Education (21YJCZH032), National Natural Science Foundation of China (No. 72104145). The fund was used in the design of the study and in the collection, analysis, and interpretation of data, as well as in the writing of the manuscript.

The original article has been corrected.

