# Supplementary material for: Weight management personas of breast cancer patients undergoing chemotherapy in China: a multi-method study
Source: BMC Med Inform Decis Mak. 2024 Apr 25;24:108. doi: 10.1186/s12911-024-02515-1 (PMC11044285; doi:10.1186/s12911-024-02515-1)
Supplement: Supplementary file 1 — Supplementary Material 1 [file 12911_2024_2515_MOESM1_ESM.docx]

**Questionnaire of Weight Management Personas of Breast Cancer Patients Undergoing Chemotherapy**

**1 Demographic**

1.1 Age: years, Time of diagnosis：＿＿＿＿＿＿

1.2 Height: m Weight: kg

1.3 Ethnicity: Han□ Others□________

1.4 Are you religious? No□ Yes□

1.5 Educational level: Primary School or below□ Middle school□ High school□ College or above □

1.6 Marital Status: Married/ In a relationship□ Single/ Divorced/ Widowed□

1.7 Resident area: City□ Town□ Village□

1.8 Employment status: Employed□ Sick leaved/unemployed□ Retired□

1.9 Family income (RMB): <3000□ 3000-5000□ 5000-10000□ ＞10000□

1.10 Health insurance: Without health insurance□ Rural program□ Citizen program□ Employee health insurance□ Others□_______

1.11 Have you used Granulocyte colony-stimulating factors (G-CSFs)? No□ Yes□

1.12 Chemotherapy：

Neoadjuvant chemotherapy□ Postoperative chemotherapy□

Chemotherapy regimen: _____________________________________________________

Date of first chemotherapy: _____________________________________________________ 1.12.1 Daily average screen time

1-2h□ 2-3h□ 3-4h□ 4-5h□ ＞5h□

**2 Persona selection**

Please read the description of five personas below carefully and choose ***no more than 2 personas*** which consistent with your own condition, not require to match the description exactly.

| **Subdomains** | **Persona A**  **Positive weight controllers** | **Persona B**  **Patients who were inactive due to fatigue** | **Persona C**  **Young patients who avoided communication** | **Persona D**  **Overweight patients with treatment priority** | **Persona E**  **Patients who engaged in irregular exercise** |
| --- | --- | --- | --- | --- | --- |
| **Representative figures** | N5-N7 | N10-N12 | N9, N16 | N2-N4, N8, N14 | N1, N13, N15 |
| **Age** | 40-50 | 44-50 | 20-30 | 39-63 | 37-47 |
| **Education level** | College or above | High school or above | College or above | High school or college | High school or college |
| **BMI** | Normal | Normal | Normal | Overweight or gained weight after diagnosis | Normal |
| **Chemotherapy cycle** | 7-8 | 2-3 | 2-6 | 7-8 | 2-8 |
| **Daily mobile phone time** | ≤4 h | 4-6 h | ＞6 h | 4-6 h | ＞6 h |
| **Perception of weight management while undergoing chemotherapy** | They are knowledgeable about weight management and are proactive in managing their weight. | They do not know about weight management. | They do not understand weight management and are afraid of knowing too much | They do not understand or know a little about weight management as a treatment priority | They know a little about weight management and control their BMI in the normal range |
| **Symptoms and emotional disturbance** | Mild symptoms: nausea, vomiting and fatigue.  No emotional issues | Severe fatigue.  No emotional issues | Nausea and vomiting.  Avoiding any kind of contact | Severe symptoms, particularly nausea.  High mental burden | Fatigue.  No emotional issues |
| **Diet and exercise change** | Regular exercise, both before and after diagnosis | Inactive due to fatigue: no exercise habit or a dislike of sports | No exercise to occasional walking | No exercise | Irregular exercise, with no exercise habits before diagnosis |
|  | High-protein diet, increased vegetable and fruit intake and a limited total calorie intake | A nutrition-focused diet with food for elevated white blood cells | Intake of more vegetables and better nutrition after diagnosis | Unrestricted diet | A high-protein diet and more vegetables and fruits |
| **Health literacy and information seeking** | Seeking knowledge actively, such as questioning rehabilitation doctors or reading professional literature | Network resources and communicate with other patients | None or just communication with other patients | Network resources and communication with other patients | Network resources and communication with other patients |

If you can't choose among the 5 personas, please write a general description of your own characteristics of weight management.

|  |
| --- |
